# Kava (*Piper methysticum* G. Forst) for Substance Use Disorders: A Review of Mechanism, Pharmacology, Clinical Evidence, and Therapeutic Potential

**DOI:** 10.3390/nu18172747

**Published:** 2026-08-22

**Authors:** Jason Krehl, Jessica Nissi Mamallapalli, Chengguo Xing, Oliver Grundmann

**Affiliations:** Department of Medicinal Chemistry, College of Pharmacy, University of Florida, Gainesville, FL 32611, USA; jason.krehl@ufl.edu (J.K.); jmamallapalli@ufl.edu (J.N.M.); chengguoxing@cop.ufl.edu (C.X.)

**Keywords:** *Piper methysticum*, kava, kavalactones, substance use disorder, alcohol use disorder, stress, anxiety, adjunctive therapy

## Abstract

Substance use disorders (SUDs) remain a major public health concern and contribute substantially to compromised quality of life, mortality, and healthcare burden. In the United States alone, millions of individuals are affected by alcohol use disorder (AUD), tobacco use disorder (TUD), and opioid use disorder (OUD), with many cases complicated by co-existing anxiety and stress-related disorders. *Piper methysticum* G. Forst (kava), a traditional South Pacific plant preparation, has gained attention for its anxiolytic, sedative, and sleep-promoting properties. Its pharmacological effects are primarily attributed to a set of lipophilic compounds known as kavalactones, which have been reported to modulate GABA_A_ receptor activity, dopaminergic and adrenergic signaling pathways, monoamine oxidase-B activity, cannabinoid receptor type 1 activity, and voltage-gated ion channels. Peer-reviewed literature was identified through searches of PubMed, NIH resources, and other scientific databases using terms related to kava, kavalactones, addiction, anxiety, stress, insomnia, and SUDs. Both clinical and preclinical studies were reviewed, including investigations of neurotransmitter systems and addiction-related signaling pathways. The current literature suggests that the strongest rationale for kava use exists in AUD, where anxiety and stress are established contributors to relapse. Evidence supporting kava use in TUD and OUD is largely theoretical, while concerns regarding hepatotoxicity, cytochrome P450 interactions, product variability, and additive risk remain important barriers to its clinical application. In summary, current evidence does not support kava as a replacement for established therapies, while its unique pharmacological profile warrants further investigation as a potential adjunctive treatment for withdrawal and relapse in SUDs.

## 1. Introduction

Substance use disorders (SUDs) continue to be a significant concern from both public health and economic perspectives. They contribute heavily to early death, impose a huge burden on the healthcare system, and could cause tremendous deterioration in quality of life among those who develop these disorders and those who provide supportive care. Some of the most prevalent disorders are alcohol use disorder (AUD), tobacco use disorder (TUD), and opioid use disorder (OUD). AUD affects millions of people worldwide and contributes to higher rates of liver and cardiovascular illness and other complications [1,2]. TUD contributes heavily to many preventable deaths from tobacco-associated illnesses such as COPD, cardiovascular diseases, stroke, and various cancers [3]. OUD has been emerging as a major public health crisis due to the increasing rates of illicit opioid use and overdose deaths [4].

Current therapeutic medications for these disorders present with varying effectiveness and often require concomitant behavior adjustment for optimal benefit. AUD is often treated with disulfiram, naltrexone, and acamprosate, which are designed to reduce cravings and prevent relapse [5]. TUD is treated via nicotine replacement therapy, varenicline or bupropion to reduce cravings, with limited effects on abstinence-associated adverse symptoms [6]. OUD is treated with opioid agonist therapies such as buprenorphine and methadone to reduce withdrawal symptoms and overdose outcomes [7,8]. Despite the available pharmacotherapy treatment options for these conditions, outcomes are often compromised by poor patient adherence, harsh side effects, and high relapse. For example, the long-term success rate of TUD is 7–9% and adverse effects include restlessness, insomnia and anxiety, which often lead to non-compliance and relapse [9]. Higher chances of successful cessation are possible if medication-assisted pharmacotherapy is accompanied by behavioral psychotherapy such as cognitive behavioral therapy [10]. Nonetheless, relapse is a serious concern for patients with SUD, and new and adjunctive therapies should be explored when and where possible.

Kava, traditionally a beverage prepared from the root of a plant (*Piper methysticum* G. Forst) known by the South Pacific Island cultures [11], has been used for its anxiolytic and stress-relieving properties in addition to sleep improvement and has gained attention throughout the whole world [12,13]. Traditionally, kava is prepared by harvesting and grinding or pounding the roots and rhizomes, mixing the material with water, and straining the resulting product. It has historically been consumed throughout the Pacific for ceremonial, social, and medicinal purposes. Modern preparations include powdered plant material, beverages, and concentrated extracts, which may differ significantly from traditional aqueous preparations in their chemical composition.

Kava is currently available in the United States as a dietary supplement to promote relaxation and can be consumed via tinctures, aqueous suspensions, capsules, and root powder. However, concerns regarding potential hepatotoxicity have contributed to regulatory scrutiny of kava internationally, including restrictions or bans in several countries including Germany, the United Kingdom, France, Canada, and Japan. Regulatory approaches have varied over time, with some restrictions subsequently being reconsidered or lifted as evidence regarding the magnitude and causality of kava-associated liver injury has evolved [14]. Despite these safety concerns, research into *P. methysticum* and its bioactive constituents has demonstrated modulation of several neurobiological targets including GABA_A_ receptor activity, dopaminergic and adrenergic signaling pathways, monoamine oxidase-B activity, cannabinoid receptor type 1 (CB1) activity, and voltage-gated ion channels [15,16,17,18,19,20]. Because of the historical use and observed beneficial effects regarding improved sleep and relaxation, kava is available in many places around the world with demand and exposure continually increasing [21].

Stress and anxiety are often cited as major triggers for alcohol and other substance use disorder relapses [22,23,24]. Many individuals with AUD report using alcohol to alleviate stress, cope with depression, or ease tension. Given the association between stress, anxiety, insomnia and SUDs, kava is a potential candidate and may be considered in combination with known pharmacotherapies. By reducing anxiety and stress, improving sleep and modulating brain reward systems, it may indirectly support recovery from SUDs, reduce relapses, and potentially even decrease total incidence.

## 2. Methods

This literature review was conducted using electronic databases of peer-reviewed literature, including but not limited to PubMed, Google Scholar, and other publicly available scientific databases, to identify relevant studies on kava and its potential use for SUD treatment. Search terms in these databases include “kava,” “kava-kava,” Piper methysticum,” “kavalactone,” “kavain,” “dihydrokavain,” “methysticin,” dihydromethysticin,” “yangonin,” “desmethoxyyangonin,” substance use disorder,” “alcohol use disorder,” “tobacco use disorder,” “opioid use disorder,” “anxiety,” “stress,” “craving,” “relapse,” “withdrawal” and combinations thereof. Studies were included if they evaluated *Piper methysticum*, kava extracts, or individual kavalactones and addressed at least one of the following topics: pharmacological mechanisms relevant to anxiety, stress, reward or addiction, pharmacokinetics or pharmacodynamics, toxicological effects or drug interactions, clinical effects on anxiety, stress, sleep, or mood relevant to substance use disorders, or direct effects on alcohol, tobacco, or opioid use disorder outcomes. Both human and preclinical studies were considered. Studies were excluded if they did not evaluate *Piper methysticum* or its major constituents, did not provide relevant pharmacological, clinical, or toxicological information, were duplicate publications, or were non-scientific sources. Studies that discussed kava solely in a historical or botanical context without any mention of pharmacology, safety, or substance use disorders were also excluded. Importantly, all studies and discussions presented in this review include various kava preparations as well as mixtures of pure kavalactones. If the composition of the kava preparation is known, it is mentioned in the discussion of the study. References to “kava” throughout this manuscript therefore refer to noble kava, other forms of kava preparations, and mixtures of synthesized or isolated kavalactones.

## 3. Results and Discussion

### 3.1. Pharmacology of Kava

**Figure 1:**
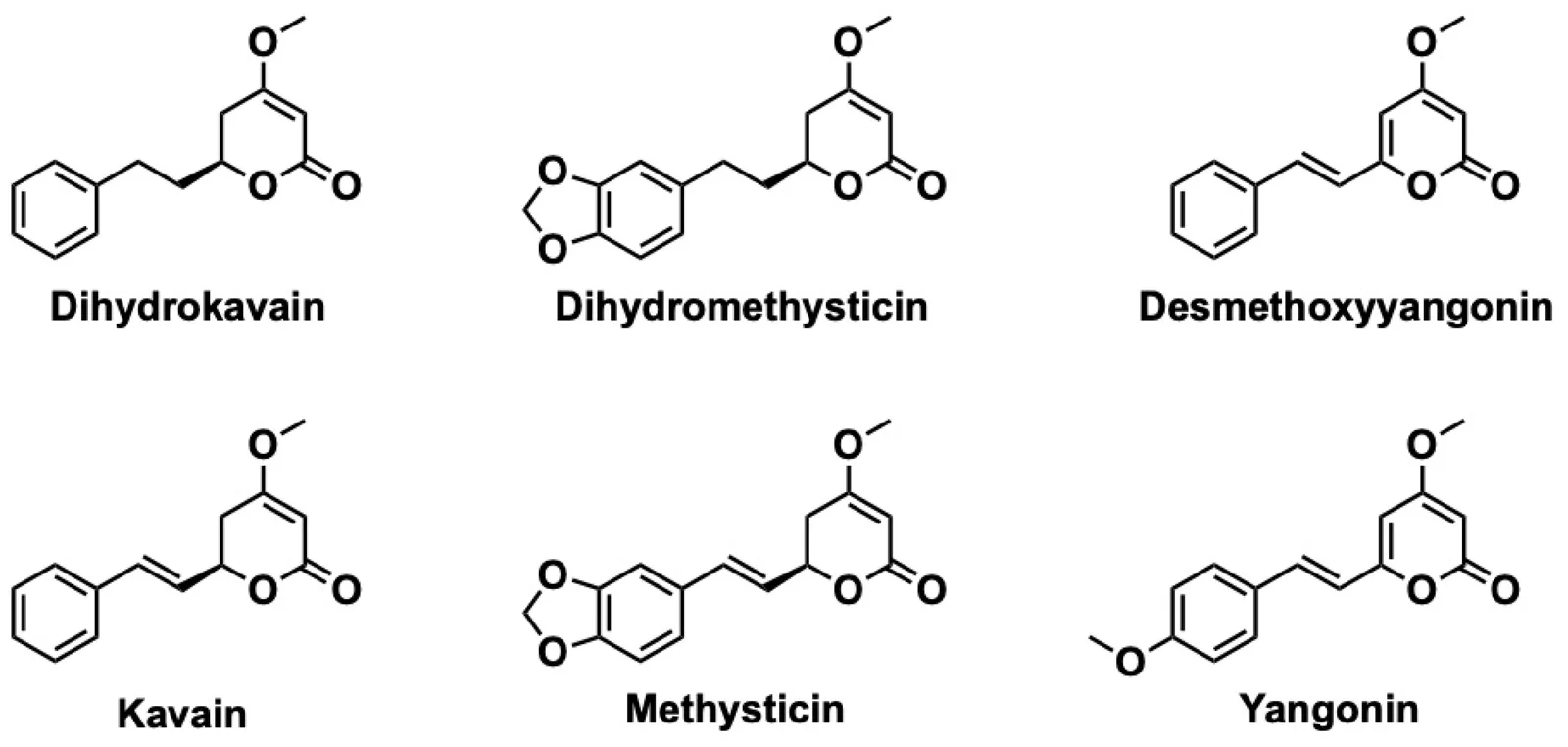
Structures of the six major pharmacologically active kavalactones.

Kava exerts its relaxing pharmacological effects mainly through a group of highly lipophilic compounds known as kavalactones, which are found in kava roots, rhizomes, and stems. Preparation of kava can derive from both roots and stems; however, the stems and stem peelings also contain a higher alkaloid content that is associated with cytotoxicity. Traditional and high-quality commercial kava derives from noble cultivars, where only the roots and rhizomes of *P. methysticum* are used [25]. While there are a large number of kavalactones identified in the kava plant, it is generally accepted that six major kavalactones are responsible for the majority of the pharmacological effects associated with kava consumption [12,26]. These compounds are kavain, dihydrokavain, methysticin, dihydromethysticin, yangonin, and desmethoxyyangonin. They differ slightly in their chemical structure (Figure 1).

A defining feature of kavalactones is their ability to penetrate the blood–brain barrier because of their lipophilic structures [12]. As shown in Table 1, predicted SwissADME profiles suggest that yangonin and desmethoxyyangonin possess greater lipophilicity and blood–brain barrier permeability compared with other major kavalactones from estimated physicochemical properties. Less lipophilic kavalactones may be responsible for the peripheral effects. However, these computational predictions remain to be experimentally tested and should not be used to guide real experimental design. Interestingly, although yangonin and desmethoxyyangonin exhibit more lipophilicity than other kavalactones, they have the least bioavailability based on human plasma analyses; dihydrokavain and dihydromethysticin have been shown to be the most bioavailable, respectively, followed by kavain, methysticin, yangonin, and desmethoxyyangonin [27]. Overall, the trend shows that kavalactones with more saturated structures are less bioavailable. Indeed, some studies indicate that despite their similar structures, these kavalactones may have distinct bioavailability properties [28,29].

Computational ADME predictions provide a beneficial initial assessment of the physicochemical characteristics that may influence individual kavalactone disposition, but they should be distinguished from experimentally determined pharmacokinetics. For example, calculated lipophilicity and gastrointestinal absorption estimates can suggest that individual kavalactones possess properties compatible with membrane penetration and absorption; however, these predictions cannot establish if a compound reaches systemic circulation, the magnitude of exposure, or the profile of the exposure in humans. These limitations are addressed by a clinical trial where five kavalactones were detectable in plasma following oral administration with maximum plasma concentrations occurring between 1 and 3 h after dosing [27]. Systemic exposure displayed dose proportionality across the range of 75–225 mg of total kavalactones. While computational modeling provides mechanistic hypotheses, human pharmacokinetic studies provide direct evidence of the systemic exposure of kavalactones and characterize the relationship between administered dose and observed plasma concentrations.

**Figure 2:**
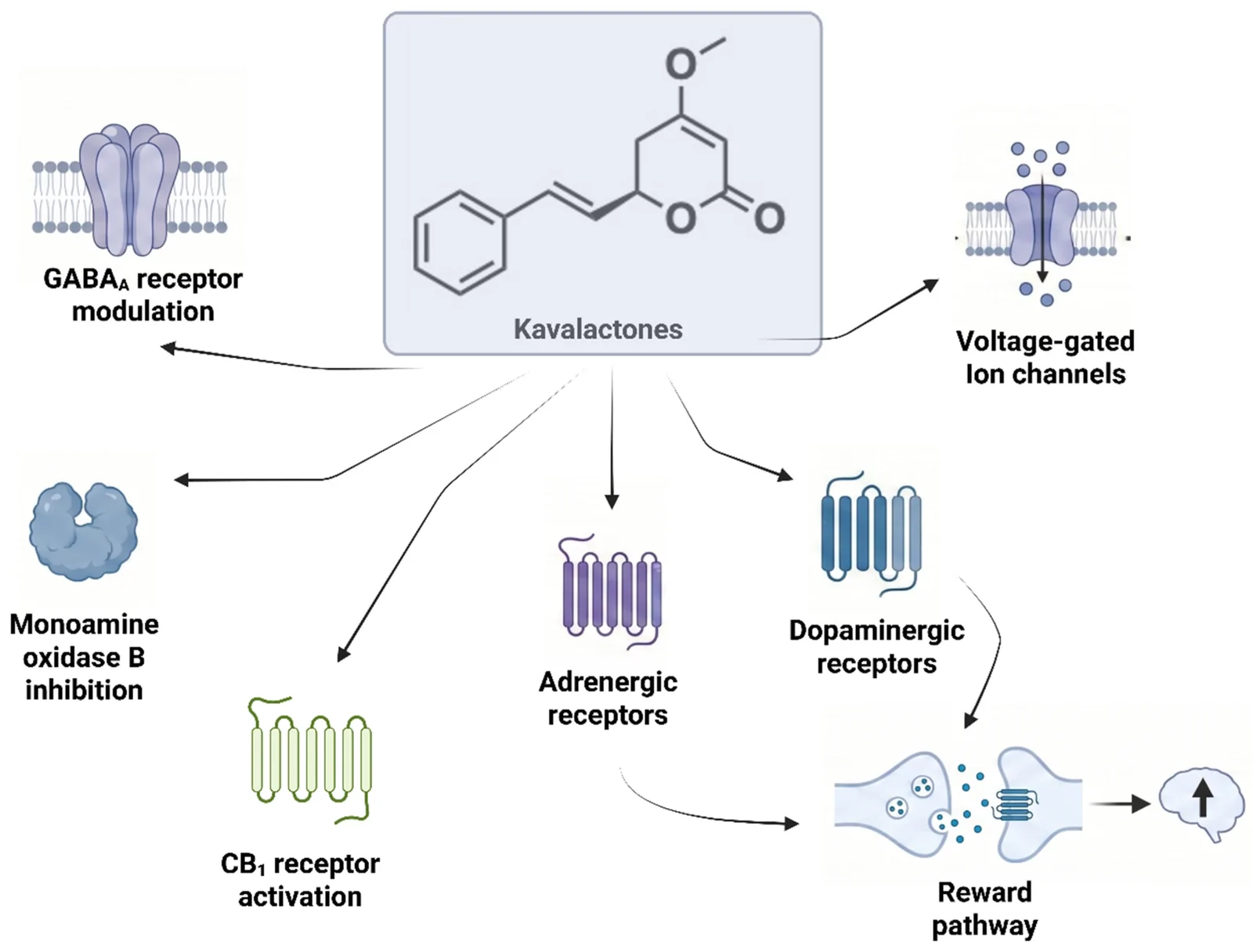
Proposed pharmacological targets of kavalactones that may contribute to their anxiolytic effects and reducing cravings and withdrawal symptoms in SUD.

Proposed mechanisms for kavalactones include modulation of GABA_A_ receptors, dopaminergic signaling, monoamine oxidase activity, cannabinoid receptor activity, and voltage-gated ion channels (Figure 2).

Although kavain is frequently cited as a major contributor to the anxiolytic effects of kava, there is currently insufficient evidence to determine which receptor systems are primarily responsible (Table 2). Kavain primarily acts by modulating the GABA_A_ receptors to increase inhibitory neurotransmission in a manner different from benzodiazepines as its action is not inhibited by the benzodiazepine antagonist flumazenil [32].

Dihydrokavain is structurally very similar to kavain and has weaker interactions at the same targets than kavain. Although its biochemical receptor interactions appear weaker, some in vivo studies suggest dihydrokavain may exhibit similar or better anxiolytic effectiveness in comparison to kavain, potentially because of its higher oral bioavailability [33,34].

Methysticin and dihydromethysticin are known for their potential to inhibit cytochrome P450, specifically CYP 1A2 and 2D6 [35]. They have been proposed to be responsible for the potential hepatotoxicity that kava may present due to their alteration of main phase 1 metabolic processes [36,37,38,39]. However, much of this inhibition has been demonstrated in vitro at concentrations well above those achieved physiologically, and emerging evidence implicates the non-kavalactone chalcones flavokavains A and B, rather than the kavalactones, as more likely contributors to the rare hepatotoxicity reported with kava [40]. Dihydromethysticin has also been shown to inhibit CYP 2B6, which can be of clinical significance [41]. While the metabolic effects are of most interest, methysticin and dihydromethysticin are also known for their connection to neuroprotective and antioxidant properties in some in vitro or pre-clinical studies [42,43]. The observation of these effects has led to theories that kavalactones may have effects that expand beyond just CNS activity and sedation. Methysticin has been shown to induce the nuclear factor erythroid 2-related factor 2 (Nrf2) and antioxidant response element to increase antioxidant enzyme expression, which may benefit against stress-induced oxidative stress in various tissues [42,44].

Yangonin and desmethoxyyangonin have a distinguishing feature as they have cannabinoid receptor type 1 activity. Yangonin has demonstrated affinity for (CB1) [20], although its activity remains substantially weaker than Δ9-tetrahydrocannabinol (THC) [45]. This interaction may partially contribute to the mood-elevating effects reported with kava use. Both yangonin and desmethoxyyangonin have been proposed to exhibit monoamine oxidase inhibitory activity in preclinical studies [17,46]. Yangonin seems to be a strong inhibitor, which could be the reason for kava’s use as a mood enhancer by preventing the break-down of serotonin and dopamine to stabilize or elevate mood. Increased circulating and synaptic levels of these neurotransmitters lead to improvements in mood (Table 2).

**Table 2:** Proposed mechanisms of action of major kavalactones, potential CNS effects, and anti-inflammatory mechanisms contributing to pharmacological spectrum of activity. ↓: reduction.

Kavalactone	Proposed Mechanisms	CNS Effects	Anti-Inflammatory Properties and Mechanism	References
Kavain	GABA_A_ modulation, sodium/calcium channel modulation	Anxiolytic, mild sedation	Moderate (↓ NF-κB, COX-2)	[19,32,47]
Dihydrokavain	Weak GABAergic activity	Mild anxiolytic	Mild–moderate cytokine reduction	[48,49,50]
Methysticin	CYP450 inhibition, antioxidant effects	Mild CNS effects	Moderate–strong (↓ iNOS, NF-κB)	[29,51]
Dihydromethysticin	CYP450 inhibition	Sedative	Moderate anti-inflammatory	[41,52]
Yangonin	CB1 receptor interaction, MAO-B inhibition	Mood elevation, euphoria	Weak–moderate	[17,20,53]
Desmethoxyyangonin	MAO-B inhibition	Mood modulation	Mild	[17,54]

It should be considered an area of further research to elucidate if the pharmacological effects of kava are a result of individual compounds or a synergistic interplay of particular compound combinations. The distinct ADME and pharmacokinetic properties of each kavalactone, despite their structural and physicochemical similarities, may further contribute to the observed poly-pharmacological effects.

An evolving area of research is uncovering the optimal kavalactone composition for providing the best therapeutic outcome while reducing side effects. Isolating individual kavalactones could be beneficial but may not be fruitful if synergism is required for effect. Individual kavalactones may not be able to reproduce the entire complex pharmacology of kava root as well. It is also possible that the variability in kavalactone composition may have differential pharmacology for different therapeutic needs.

Several placebo-controlled trials have been conducted that investigate kava’s efficacy in individuals with generalized anxiety disorder (GAD) [55,56,57,58,59,60]. Earlier studies, primarily from Europe, have reported on the benefits of kava in the management of mild to moderate anxiety. In the kava anxiety depression spectrum study (KADSS), a randomized placebo-controlled trial with 60 volunteers that experienced high levels of anxiety reported that kava extract use significantly reduced their symptoms [54] as compared to placebo. These results contrast with a follow-up study that was performed as a 16-week phase-three trial with 171 volunteers, all of whom were previously diagnosed with GAD, that found no differences [56] between the placebo and the kava group. In additional contrast, a more recent study from Australia reported no significant anxiolytic effects, highlighting inconsistency in the clinical evidence base [56]. Overall, kava has not been adopted as a standard pharmacological treatment for GAD, and its therapeutic role remains limited to mild anxiety contexts with mixed empirical support. Differences in extract composition, dosing regimens, participant anxiety severity, and placebo responsiveness may partially explain the conflicting findings observed across clinical trials [55,56,57,61,62,63].

### 3.2. Kava and Alcohol Use Disorder (AUD)

Alcohol is the most commonly used intoxicant substance worldwide and accounts for 6% of deaths worldwide [64]. Thousands of US adults die from alcohol poisoning every year, and over 14 million US adults suffer from AUD [65], with a 20% increase in mortality in persons with AUD during the COVID-19 pandemic [66]. Between 2011 and 2015, on average 255 lives were lost per day due to excessive alcohol use, with over $249 billion loss in productivity in the US alone [67]. In addition to the high mortality associated with AUD, there is also a discrepancy between rural and urban counties in the US, with rural areas experiencing a higher mortality rate of 15.8 deaths compared to 12.7 deaths per 100,000 [68].

Mechanistically, chronic alcohol use alters dopamine signaling pathways and increases sensitivity to anxiety and withdrawal symptoms during abstinence [24,35,39,69]. High anxiety levels are associated with an increase in cravings for alcohol and, therefore, higher relapse rates as shown in pre-clinical animal and clinical trials [70,71,72]. The reciprocal relationship between anxiety and AUD identifies a need for research into compounds that can utilize their anxiolytic properties to reduce and prevent the need for increasing or excessive alcohol consumption and withdrawal symptoms. This has been shown with the use of antidepressants and anxiolytics in patients in AUD remission [71,73,74,75,76,77].

Direct preclinical evidence of kava in managing AUD is sparse, but the pharmacological properties described earlier provide a reasonable mechanistic basis for further research to be conducted. In terms of clinical evidence, there is currently no clinical trial that has evaluated kava as treatment for reducing AUD relapses or cravings. Most kava clinical studies are specifically on anxiolytic properties, which may or may not be effective for reducing alcohol abuse while participants were recommended to restrain from alcohol use during those trials.

At the same time, many users report that their alcohol consumption has gone down while using kava recreationally because of the substitution effect, or anxiety-related reasons for drinking being curbed [78,79]. Despite this, targeted studies that examine specific metrics regarding alcohol use, such as length of abstinence, total alcohol intake, or craving and withdrawal symptom severity, are not robust. Current evidence suggests that the potential benefits of kava in AUD are more likely from anxiolytic and stress-reducing effects rather than a direct solution to cravings or addiction.

A recent survey among kava bar patrons reported that half (51.7% or 93 of 180) of respondents conceptualized kava as an alcohol substitute or replacement [78]. The survey also supported many of the clinical study findings of kava reducing anxiety, improving mood, and promoting relaxation and sedation.

It is important to make note of the speculative nature of these studies and their conclusions due to the lack of specific, targeted alcohol use disorder clinical trials for kava. Given the current lack of direct benefits, kava should be considered as an adjunctive or add-on treatment to known pharmacotherapies such as naltrexone, to be used for anxiety-related relapse triggers that current medication does not address. At the same time, because both kava and chronic alcohol use carry hepatotoxic potential, combined use would require additional caution in patients with heavy alcohol consumption or pre-existing liver disease, who may need to be excluded from kava use altogether.

### 3.3. Kava and Tobacco Use Disorder (TUD)

With continuing efforts over the past few decades, cigarette use in the US has declined substantially. Such a reduction, however, led to misperception that cigarette use is no longer a critical pandemic, partly due to variations in its prevalence in different communities [80,81,82]. Indeed, cigarette use is still highly prevalent and persistent in the US, with 28 million adult smokers [83,84] and no indication of a significant reduction in the near future as ~1500 American youth start their first cigarette each day [85]. About 50% of smokers would die of smoking-related diseases, resulting in $170 billion direct medical costs [86] and >$800 billion total lost productivity each year in the US [87]. Despite the availability of FDA-approved cessation medications, <10% smokers achieve abstinence even after multiple attempts largely because current cessation medications fail to address abstinence-associated adverse symptoms (stress, anxiety and insomnia) which drive relapse [9]. The FDA and NIH therefore have emphasized the need for “next-generation” cessation therapies to alleviate abstinence-associated adverse symptoms and reduce cigarette smoke-caused harms [9].

The rationale for investigating kava as an alternative or adjunct treatment in TUD stems from its anxiolytic pathways and dopamine signaling, both of which directly contribute to nicotine dependence. Nicotine produces its effects by activating nicotinic acetylcholine receptors, specifically α4β2 subtype receptors, which releases dopamine [46,69] and causes smoking habits to be reinforced. Kavalactones, the main compounds in kava, have been studied to influence many systems related to addiction, most notably dopaminergic and GABAergic systems. Some preclinical studies suggest that kavalactones may influence dopamine reuptake and GABAergic signaling pathways associated with addiction and stress response [36,38,46]. Indeed, kava is currently consumed by some smokers in the US to manage tobacco dependence with ~75% satisfaction, based on a recent kava survey [88]. A 4-week cigarette smoke mouse study indicated kava’s holistic potential in managing cigarette use—alleviating somatic withdrawal symptoms, lowering cigarette smoke-induced stress hormones, modulating CREB phosphorylation in the cerebellum, reducing anxiety, suppressing cigarette smoke-induced lung inflammation with a potential to improve lung functions [89,90], and thus addressing key limitations of current cessation medications.

Currently, no randomized controlled trials demonstrate the efficacy of kava for smoking cessation or relapse prevention, but pharmacology and mechanisms provide a reasonable basis for investigating its potential for reducing nicotine cravings and stress-related relapses. One clinical trial is ongoing, assessing the potential of kava to facilitate smoking cessation, focusing on its potential to address abstinence-associated adverse symptoms—stress, anxiety and insomnia [91].

It is plausible that kava may function as a behavioral substitute for tobacco use. Smoking is closely tied to social routines, relaxation, and stress relief, often embedded within broader ritualized contexts that may also include alcohol use [41]. Similarly, kava has a long history of social and ceremonial use to promote relaxation, aligning with some of the reinforcing psychosocial aspects associated with tobacco consumption. Key research gaps remain, particularly the absence of trials assessing standardized dosing, cessation outcomes (e.g., abstinence duration, relapse rates, withdrawal severity), and interactions with established nicotine replacement therapies. Safety considerations, including long-term use and hepatotoxicity risk, also require clarification. Future work should further distinguish whether any potential benefit is driven primarily by behavioral substitution, anxiolytic effects, or direct modulation of addiction-related neurotransmitter pathways. Given the high relapse rates in TUD and the need for alternative or adjunctive approaches, further investigation into kava remains mechanistically and clinically relevant.

### 3.4. Kava and Opioid Use Disorder (OUD)

The ongoing opioid epidemic accounts for more than 40,000 deaths per year since 2017 [92]. As of 2017, an estimated 2.1 million US persons aged 12 and older were diagnosed with OUD, leading to an overall economic burden of $1.02 trillion in the US alone [93].

The potential role of kava in managing OUD is largely theoretical and based on its anxiolytic and sedative properties rather than any direct interaction with opioid receptors. The discussed kavalactones may reduce stress-related triggers for opioid relapse, but do not address the core neurochemical pathways targeted by established therapies.

Unlike methadone and buprenorphine, which have strong evidence for reducing withdrawal, cravings, relapse, and mortality, kava has not been studied in clinical trials for OUD. Any potential benefit would likely be limited to adjunctive stress or anxiety reduction rather than treatment of dependence itself, and there is currently no evidence that it improves clinical outcomes such as abstinence or relapse prevention.

Important safety concerns also limit its application, including potentially additive CNS depression when combined with opioids, benzodiazepines, or alcohol, with reported increased risks of sedation and respiratory depression [94,95].

Overall, the current evidence does not support kava as primary or adjunctive treatment for OUD. While its pharmacology provides a rationale for further investigation as a potential adjunct for stress modulation, its role remains theoretical pending controlled clinical studies addressing efficacy, safety, and mechanism of action.

### 3.5. Safety and Toxicology

Concerns regarding kava’s potential hepatotoxicity have influenced the regulatory and clinical evaluation of kava and kava products. Kava has been consumed for centuries in the Pacific Island region with few reports of hepatotoxicity. Most of the reported hepatotoxic cases come from North America and Europe in the early 2000s in association with the anxiolytic form of kava, which was typically an acetone or ethanolic extraction of kava instead of the aqueous traditional preparation, with severe cases resulting in total liver failure [39]. The current consensus is that traditional kava in the beverage form is generally safe and the hepatotoxic risk is extremely rare and potentially idiosyncratic. Multiple mechanisms have been proposed with no validation, including the contamination of the raw materials with aflatoxins, the inclusion of non-root plant parts (leaves and/or stems), the inclusion of kava cultivars not recommended for traditional kava consumptions (Tudei kava, which can cause sickness for 2 days and contains higher levels of flavokavains A and B) instead of noble kava, the use of organic extraction instead of traditional aqueous preparation, and the potential modulations of CYP 3A4 and 2D6 enzymes to induce the risk of drug–herb interaction [35,36,38]. However, controlled human studies by Gurley and colleagues found that kava does not inhibit CYP3A4 or CYP2D6 in a clinically impactful manner and produces a meaningful reduction only in CYP2E1 [96]. As noted above, the non-kavalactone flavokavains A and B have emerged as more probable contributors to the rare cases of kava-associated hepatotoxicity.

Kava may interact both pharmacodynamically and pharmacokinetically with many classes of central nervous system-active and other drugs that undergo extensive metabolism. Because of the effects kava produces in humans, it is likely that, when combined with other CNS-active drugs, such as benzodiazepines or alcohol, an additive or synergistic effect may be produced, resulting in further sedation. Some studies have found significant impairments in motor function in those who combined alprazolam with kava products [35,36,94,95].

Other factors to consider are the dosage, duration of use, preexisting liver conditions, and the quality of the kava preparation. Due to various aqueous suspensions, tinctures, root powders, and capsules available to consumers, these products contain varying amounts of kavalactones per recommended dose, resulting in major kavalactone exposure differences. Kavalactone abundance in product labels has been shown to differ up to 90% from experimental measurements, highlighting the need for rigorous quality control of kava products to more accurately comprehend the pharmacology and safety [63]. It remains debated whether hepatotoxicity is primarily associated with nontraditional extraction methods, stem and leaf, cultivars containing high levels of flavokavains A and B, contamination, or metabolic bioactivation rather than traditional aqueous root preparations alone [36,39,97]. Because of these uncertainties, Germany reversed its ban in 2014 after the scientific literature re-examined kava’s safety profile.

Currently, kava is regulated differently across the world. In the United States, kava is classified as a dietary supplement, and advisories on kava products and their potential for hepatotoxicity have been released. Rigorous standardization would benefit consumers where kava products are derived from noble cultivars where only the roots and rhizomes are used to reflect the traditional preparation and use of kava. European nations still impose restrictions on the sale of kava and tightly regulate what label claims can be made about kava, specifically regarding therapeutic claims. Australia, on the other hand, has recently classified kava in the food category as a pilot program.

### 3.6. Potential Role as Adjunct Therapy and Future Research Directions

Kava should not be considered a replacement for evidence-based treatments for SUDs, but rather as a potential adjunctive intervention that may complement existing approaches. Current evidence suggests that kava’s anxiolytic and stress-relieving properties can be clinically relevant in groups where anxiety and stress are the main drivers of substance abuse.

A promising application of kava may be in individuals who have alcohol or tobacco use disorders and concomitant anxiety-related disorders, as anxiety symptoms are prevalent in individuals with alcohol use disorder, and the presence of both results in worse treatment outcomes. Because kava demonstrates anxiolytic effects without cognitive impairment [57,61,62], it has been proposed that it could serve as an alternative for managing anxiety symptoms compared to benzodiazepines, which produce heavier sedation. By reducing baseline anxiety, kava may indirectly lower the craving for alcohol as a form of self-medication.

Kava may be able to function as an early intervention during recovery periods or also during periods of higher risk when an individual experiences high stress levels.

Although interest in kava as a possible treatment for SUDs has grown in recent years, many important questions remain unanswered. One of the most important next steps is the development of randomized controlled trials (RCTs) comparing kava either directly to placebo or as an adjunct to standard pharmacotherapy in patients with alcohol or tobacco use disorder. Current evidence suggests that kava may help reduce anxiety, stress, and substance cravings, but many studies have been limited by small sample sizes, inconsistent dosing methods, and minimal follow-up studies. Future clinical trials should examine outcomes such as relapse rates, withdrawal symptoms, craving intensity, anxiety reduction, and overall quality of life to better determine whether kava could serve as a meaningful adjunct treatment for alcohol or tobacco use disorder in patients with concomitant anxiety disorders.

Future randomized controlled trials should use standardized kava preparations with a defined total kavalactone content and clearly characterized individual kavalactone composition. Previous clinical studies evaluating kava for anxiety have administered approximately 12–140 mg/day of total kavalactones, while recent pharmacokinetic research has evaluated single doses of 75–225 mg and repeated dosing of 75 mg three times daily [27,56]. These studies provide reasonable starting ranges for dose-finding studies, but establishing doses for anxiety should not be assumed to produce equivalent effects on substance use disorder outcomes. Future AUD, TUD, and OUD trials should incorporate dose range studies before defining a fixed therapeutic dose.

Dose–response relationships for kavalactones also remain poorly understood. Existing studies vary considerably in extract type, kavalactone concentration, and dosing regimens, making it difficult to determine the optimal balance between therapeutic benefit and adverse effects. Future research should examine both the pharmacological and clinical effects of chemically well-characterized kava preparations while also evaluating long-term use, tolerance development, and potential interactions with alcohol, benzodiazepines, antidepressants, and other centrally acting substances frequently used in patients with SUDs.

Overall, further research is needed to better define kava’s therapeutic role, safety profile, and long-term clinical value. With more rigorous and standardized investigation, kava may emerge as a useful complementary approach in addiction medicine and the treatment of anxiety-related SUDs.

## 4. Conclusions

Kava (*Piper methysticum*) occupies an interesting but still early stage of investigation within addiction medicine. Across the literature, its most consistent and well-supported effects relate to anxiety reduction and stress modulation, which are potentially driven by a broad range of pharmacological actions involving GABA_A_ receptor activity [15,16,29,32,33,34], dopaminergic signaling [46,69], monoamine oxidase inhibition [17,46], cannabinoid receptor interactions [20], and ion channel effects [19]. When considered together, these proposed mechanisms help explain why kava has attracted attention as a potential supportive agent in conditions where stress and emotional dysregulation play a major role in substance use behavior. The primary caveat is that rigorous clinical trials need to be conducted with chemically well-defined kava products to determine to what degree kava can benefit patients with a SUD. However, more pre-clinical evidence is still needed to support kava’s potential use in aiding AUD, TUD, and OUD, while the evidence for AUD is strongest yet still limited.

At present, the most investigated application for kava appears to be in alcohol use disorder. This is not because kava directly reduces alcohol reinforcement, but rather because it may reduce anxiety and stress, which commonly trigger drinking and relapse. However, the clinical evidence remains limited and somewhat inconsistent, and there are no strong, large-scale randomized controlled trials that directly measure outcomes like relapse prevention or sustained reduction in alcohol use.

When it comes to tobacco and opioid use disorders, there is to date no clinical evidence supporting its adjunctive use. Most investigations are theoretical or preclinical, with some mechanistic overlap suggesting possible effects on dopamine and stress pathways, but very little direct clinical data. Because of this, there is currently no clinical evidence to suggest kava has a meaningful role in reducing cravings, withdrawal severity, or relapse risk in these populations. However, preclinical studies indicate a potential benefit in tobacco use disorder that needs further investigation. In contrast, established treatments such as nicotine replacement therapy, varenicline, buprenorphine, and methadone remain the standard of care.

Safety remains one of the biggest limiting factors in considering kava for clinical use. Concerns around hepatotoxicity are still not fully resolved and appear to involve multiple possible mechanisms, including metabolic activation, CYP450 interactions, and variability in plant material and extraction methods. In addition, kava’s sedative properties raise concerns about additive effects when combined with alcohol, opioids, benzodiazepines, or other central nervous system depressants. The lack of standardization across commercial products only adds to this uncertainty and complexity. Specifically, there are a wide range of kava products on the market with varied chemical compositions and thus safety profiles and biological characteristics. They should not be considered the same nor treated equally: if one kava product is effective for one indication, it does not mean that other kava products will be equally effective; similarly, if one kava product is not safe, it does not mean other kava products are not safe either. It should also be emphasized that “Dose makes a poison”—kava consumers need to follow the proper dose and regimen because more is not always better, including its benefits. Overall, kava should not be viewed as a replacement for evidence-based treatments in SUDs because of a lack of clinically rigorous trials. However, its anxiolytic properties and relatively unique pharmacology make it worth continuing investigation as a possible adjunctive option, particularly for individuals whose substance use is strongly associated with stress and anxiety. Moving forward, well-designed clinical trials, chemically defined kava formulations, and clear safety data will be necessary before kava’s role in addiction treatment can be properly defined.

## Figures and Tables

**Table 1:** SwissADME predictions generated using the Swiss Institute of Bioinformatics SwissADME platform. Predicted pharmacokinetic and physicochemical properties of the major kavalactones based on SwissADME prediction and the experimental published literature. Increased lipophilicity may contribute to enhanced blood–brain barrier penetration and central nervous system activity.

Compound	MW (g/mol)	Predicted Log P	Predicted GI Absorption	Experimental % Uptake CaCO-2 ^1^	Predicted BBB Permeability	Experimental BBB Penetration	References
Kavain	230.26	2.8–3.1	High	80%	Yes	Yes	[30,31]
Dihydrokavain	232.28	2.5–2.9	High	83%	Yes	Yes	[30,31]
Methysticin	274.27	2.3–2.7	High	74%	Yes	No	[31]
Dihydromethysticin	276.28	2.1–2.6	High	71%	Yes	No	[31]
Yangonin	258.27	3.5–4	High	35%	Yes	Yes	[30,31]
Desmethoxyyangonin	228.24	3.2–3.7	High	66%	Yes	Yes	[30,31]

^1^ Apical to basolateral % transport of total amount dissolved in water after 90 min.

## Data Availability

No new data were created or analyzed in this study. Data sharing is not applicable to this article.

## References

[1] World Health Organization (2024). Global Status Report on Alcohol and Health and Treatment of Substance Use Disorders.

[2] Rehm J., Samokhvalov A.V., Shield K.D. (2013). Global burden of alcoholic liver diseases. J. Hepatol..

[3] U.S. Department of Health and Human Services (2014). The Health Consequences of Smoking—50 Years of Progress: A Report of the Surgeon General.

[4] Volkow N.D., Blanco C. (2021). The changing opioid crisis: Development, challenges and opportunities. Mol. Psychiatry.

[5] Heilig M., Goldman D., Berrettini W., O’Brien C.P. (2011). Pharmacogenetic approaches to the treatment of alcohol addiction. Nat. Rev. Neurosci..

[6] Cahill K., Stevens S., Lancaster T. (2014). Pharmacological treatments for smoking cessation. JAMA.

[7] Mattick R.P., Breen C., Kimber J., Davoli M. (2014). Buprenorphine maintenance versus placebo or methadone maintenance for opioid dependence. Cochrane Database Syst. Rev..

[8] Administration SA and MHS (2021). TIP 63: Medications for Opioid Use Disorder.

[9] Warraich H.J., King B.A., Compton W.M., Herrmann E.S., Hai M.T., Califf R.M., Bertagnolli M.M. (2025). Opportunities for innovation in smoking cessation therapies: A perspective from the National Institutes of Health and US Food and Drug Administration. Ann. Intern. Med..

[10] Perry C., Liberto J., Milliken C., Burden J., Hagedorn H., Atkinson T., McKay J.R., Mooney L., Sall J., Sasson C. (2022). The management of substance use disorders: Synopsis of the 2021 US Department of Veterans Affairs and US Department of Defense clinical practice guideline. Ann. Intern. Med..

[11] WFO *Piper methysticum* G. Forst. World Flora Online. http://www.worldfloraonline.org/taxon/wfo-0000486456.

[12] Vincent L., Mark M., Lamont L. (1997). Kava: The Pacific Elixir: The Definitive Guide to Its Ethnobotany, History, and Chemistry.

[13] Bilia A.R., Gallon S., Vincieri F.F. (2002). Kava-kava and anxiety: Growing knowledge about the efficacy and safety. Life Sci..

[14] Economidis G., Lynch M., Taylor S., Asare-Doku W., Macpherson G., Degenhardt L., Farrell M., Santo T. (2025). Global Perspectives on Kava: A Narrative Systematic Review of the Health Effects, Economic and Social Impacts and Policy Considerations. Drug Alcohol Rev..

[15] Davies L.P., Drew C.A., Duffield P., Johnston G.A.R., Jamieson D.D. (1992). Kava pyrones and resin: Studies on GABAA, GABAB and benzodiazepine binding sites in rodent brain. Pharmacol. Toxicol..

[16] Jussofie A., Schmiz A., Hiemke C. (1994). Kavapyrone enriched extract from *Piper methysticum* as modulator of the GABA binding site in different regions of rat brain. Psychopharmacology.

[17] Prinsloo D., van Dyk S., Petzer A., Petzer J.P. (2019). Monoamine oxidase inhibition by kavalactones from kava (*Piper methysticum*). Planta Med..

[18] Cairney S., Maruff P., Clough A.R. (2002). The neurobehavioural effects of kava. Aust. N. Z. J. Psychiatry.

[19] Schirrmacher K., Büsselberg D., Langosch J.M., Walden J., Winter U., Bingmann D. (1999). Effects of (±)-kavain on voltage-activated inward currents of dorsal root ganglion cells from neonatal rats. Eur. Neuropsychopharmacol..

[20] Ligresti A., Villano R., Allarà M., Ujváry I., Di Marzo V. (2012). Kavalactones and the endocannabinoid system: The plant-derived yangonin is a novel CB1 receptor ligand. Pharmacol. Res..

[21] Bian T., Corral P., Wang Y., Botello J., Kingston R., Daniels T., Salloum R.G., Johnston E., Huo Z., Lu J. (2020). Kava as a Clinical Nutrient: Promises and Challenges. Nutrients.

[22] Spanagel R. (2003). Alcohol addiction research: From animal models to clinics. Best Pract. Res. Clin. Gastroenterol..

[23] Anthenelli R.M. (2012). Overview: Stress and alcohol use disorders revisited. Alcohol. Res..

[24] Koob G.F., Volkow N.D. (2016). Neurobiology of addiction: A neurocircuitry analysis. Lancet Psychiatry.

[25] World Health Organization (2007). Assessment of the Risk of Hepatotoxicity with Kava Products.

[26] Singh Y.N. (2004). An Introduction to Kava *Piper methysticum*. Kava: From Ethnology to Pharmacology.

[27] Kanumuri S.R.R., Mamallapalli J., Nelson R., McCurdy C.R., Mathews C.A., Xing C., Sharma A. (2022). Clinical pharmacokinetics of kavalactones after oral dosing of standardized kava extract in healthy volunteers. J. Ethnopharmacol..

[28] Wang Y., Eans S.O., Stacy H.M., Narayanapillai S.C., Sharma A., Fujioka N., Haddad L., McLaughlin J., Avery B.A., Xing C. (2018). A stable isotope dilution tandem mass spectrometry method of major kavalactones and its applications. PLoS ONE.

[29] Dinh L.D., Simmen U., Bueter K.B., Bueter B., Lundstrom K., Schaffner W. (2001). Interaction of various *Piper methysticum* cultivars with CNS receptors in vitro. Planta Med..

[30] Keledjian J., Duffield P.H., Jamieson D.D., Lidgard R.O., Duffield A.M. (1988). Uptake into mouse brain of four compounds present in the psychoactive beverage kava. J. Pharm. Sci..

[31] Matthias A., Blanchfield J.T., Penman K.G., Bone K.M., Toth I., Lehmann R.P. (2007). Permeability studies of Kavalactones using a Caco-2 cell monolayer model. J. Clin. Pharm. Ther..

[32] Chua H.C., Christensen E.T.H., Hoestgaard-Jensen K., Hartiadi L.Y., Ramzan I., Jensen A.A., Absalom N.L., Chebib M. (2016). Kavain, the major constituent of the anxiolytic kava extract, potentiates GABAA receptors: Functional characteristics and molecular mechanism. PLoS ONE.

[33] Smith K.K., Dharmaratne H.R.W., Feltenstein M.W., Broom S.L., Roach J.T., Nanayakkara N.P., Khan I.A., Sufka K.J. (2001). Anxiolytic effects of kava extract and kavalactones in the chick social separation-stress paradigm. Psychopharmacology.

[34] Feltenstein M.W., Lambdin L.C., Ganzera M., Ranjith H., Dharmaratne W., Nanayakkara N.P.D., Khan I.A., Sufka K.J. (2003). Anxiolytic properties of *Piper methysticum* extract samples and fractions in the chick social–separation–stress procedure. Phytother. Res..

[35] Mathews J.M., Etheridge A.S., Black S.R. (2002). Inhibition of human cytochrome P450 activities by kava extract and kavalactones. Drug Metab. Dispos..

[36] Clouatre D.L. (2004). Kava kava: Examining new reports of toxicity. Toxicol. Lett..

[37] Johnson B.M., Qiu S.-X., Zhang S., Zhang F., Burdette J.E., Yu L., Bolton J.L., van Breemen R.B. (2003). Identification of novel electrophilic metabolites of *Piper methysticum* Forst. (Kava). Chem. Res. Toxicol..

[38] Teschke R., Schulze J. (2010). Risk of kava hepatotoxicity and the FDA consumer advisory. JAMA.

[39] Teschke R. (2010). Kava hepatotoxicity. A clinical review. Ann. Hepatol..

[40] Teschke R., Qiu S.X., Lebot V. (2011). Herbal hepatotoxicity by kava: Update on pipermethystine, flavokavain B, and mould hepatotoxins as primarily assumed culprits. Dig. Liver Dis..

[41] Wang P.-F., Xing C., Kharasch E.D. (2025). Inhibition of cytochrome P450 2B6 activity by dihydromethysticin: Structural and mechanistic insights. Drug Metab. Dispos..

[42] Dittmar M.C., Tohidnezhad M., Fragoulis A., Bücker A., Stein M., Pufe T., Kubo Y. (2024). Pharmacological effects of methysticin and L-sulforaphane through the Nrf2/ARE signaling pathway in MLO-Y4 osteocytes: In vitro study. Ann. Anat.-Anat. Anz..

[43] Fragoulis A., Siegl S., Fendt M., Jansen S., Soppa U., Brandenburg L.-O., Pufe T., Weis J., Wruck C.J. (2017). Oral administration of methysticin improves cognitive deficits in a mouse model of Alzheimer’s disease. Redox Biol..

[44] Wruck C.J., Götz M.E., Herdegen T., Varoga D., Brandenburg L.-O., Pufe T. (2008). Kavalactones protect neural cells against amyloid β peptide-induced neurotoxicity via extracellular signal-regulated kinase 1/2-dependent nuclear factor erythroid 2-related factor 2 activation. Mol. Pharmacol..

[45] Sinha R. (2008). Chronic stress, drug use, and vulnerability to addiction. Ann. N. Y. Acad. Sci..

[46] Wise R.A. (1981). Brain dopamine and reward. Theory Psychopharmacol..

[47] Chen X., Qin X., Bai W., Ren J., Yu Y., Nie H., Li X., Liu Z., Huang J., Li J. (2024). Kavain alleviates choroidal neovascularization via decreasing the activity of the HIF-1α/VEGF-A/VEGFR2 signaling pathway and inhibiting inflammation. Adv. Pharm. Bull..

[48] Yuan C.-S., Dey L., Wang A., Mehendale S., Xie J.-T., Aung H.H., Ang-Lee M.K. (2002). Kavalactones and dihydrokavain modulate GABAergic activity in a rat gastric-brainstem preparation. Planta Med..

[49] Boonen G., Häberlein H. (1998). Influence of genuine kavapyrone enantiomers on the GABAA binding site. Planta Med..

[50] Folmer F., Blasius R., Morceau F., Tabudravu J., Dicato M., Jaspars M., Diederich M. (2006). Inhibition of TNFα-induced activation of nuclear factor κB by kava (*Piper methysticum*) derivatives. Biochem. Pharmacol..

[51] Fu S., Rowe A., Ramzan I. (2012). Kavalactone metabolism in rat liver microsomes. Phytother. Res..

[52] Wu D., Yu L., Nair M.G., DeWitt D.L., Ramsewak R.S. (2002). Cyclooxygenase enzyme inhibitory compounds with antioxidant activities from *Piper methysticum* (kava kava) roots. Phytomedicine.

[53] Chow L.-H., Lin P.-C., Chen Y.-J., Chen Y.-H., Huang E.Y.-K. (2024). Yangonin, one of the kavalactones isolated from *Piper methysticum* G. Forst, acts through cannabinoid 1 (CB1) receptors to induce an intrathecal anti-hyperalgesia. J. Ethnopharmacol..

[54] Uebelhack R., Franke L., Schewe H.-J. (1998). Inhibition of platelet MAO-B by kava pyrone-enriched extract from *Piper methysticum* Forster (kava-kava). Pharmacopsychiatry.

[55] Sarris J., Kavanagh D.J., Byrne G., Bone K.M., Adams J., Deed G. (2009). The Kava Anxiety Depression Spectrum Study (KADSS): A randomized, placebo-controlled crossover trial using an aqueous extract of *Piper methysticum*. Psychopharmacology.

[56] Sarris J., Byrne G.J., Bousman C.A., Cribb L., Savage K.M., Holmes O., Murphy J., Macdonald P., Short A., Nazareth S. (2020). Kava for generalised anxiety disorder: A 16-week double-blind, randomised, placebo-controlled study. Aust. N. Z. J. Psychiatry.

[57] Connor K.M., Davidson J.R.T. (2002). A placebo-controlled study of Kava kava in generalized anxiety disorder. Int. Clin. Psychopharmacol..

[58] Connor K.M., Payne V., Davidson J.R.T. (2006). Kava in generalized anxiety disorder: Three placebo-controlled trials. Int. Clin. Psychopharmacol..

[59] Cribb L., Sarris J., Savage K.M., Byrne G.J., Metri N.-J., Scholey A., Stough C., Bousman C.A. (2023). Effect of kava (*Piper methysticum*) on peripheral gene expression among individuals with generalized anxiety disorder: A post hoc analysis of a randomized controlled trial. Phytother. Res..

[60] Boerner R.J., Sommer H., Berger W., Kuhn U., Schmidt U., Mannel M. (2003). Kava-Kava extract LI 150 is as effective as Opipramol and Buspirone in Generalised Anxiety Disorder–an 8-week randomized, double-blind multi-centre clinical trial in 129 out-patients. Phytomedicine.

[61] Sarris J., Stough C., Bousman C.A., Wahid Z.T., Murray G., Teschke R., Savage K.M., Dowell A., Ng C., Schweitzer I. (2013). Kava in the treatment of generalized anxiety disorder: A double-blind, randomized, placebo-controlled study. J. Clin. Psychopharmacol..

[62] Pittler M.H., Ernst E. (2003). Kava extract versus placebo for treating anxiety. Cochrane Database Syst. Rev..

[63] Mamallapalli J., Kanumuri S.R.R., Corral P., Johnston E., Zhuang C., McCurdy C.R., Mathews C.A., Sharma A., Xing C. (2022). Characterization of different forms of kava (*Piper methysticum*) products by UPLC-MS/MS. Planta Med..

[64] Strayer R.J., Friedman B.W., Haroz R., Ketcham E., Klein L., LaPietra A.M., Motov S., Repanshek Z., Taylor S., Weiner S.G. (2023). Emergency department management of patients with alcohol intoxication, alcohol withdrawal, and alcohol use disorder: A white paper prepared for the American Academy of Emergency Medicine. J. Emerg. Med..

[65] Cohen S.M., Alexander R.S., Holt S.R. (2022). The spectrum of alcohol use: Epidemiology, diagnosis, and treatment. Med. Clin..

[66] White A.M., Castle I.-J.P., Powell P.A., Hingson R.W., Koob G.F. (2022). Alcohol-related deaths during the COVID-19 pandemic. JAMA.

[67] Esser M.B. (2020). Deaths and years of potential life lost from excessive alcohol use—United States, 2011–2015. MMWR Morb. Mortal. Wkly. Rep..

[68] Spencer M.R., Curtin S.C., Garnette M.F. (2022). *Quickstats*: Age-adjusted rates* of alcohol-induced deaths, ^†^ by urban-rural status ^§^—United States, 2000–2020. MMWR Morb. Mortal. Wkly. Rep..

[69] Volkow N.D., Fowler J.S., Wang G.-J., Swanson J.M., Telang F. (2007). Dopamine in drug abuse and addiction: Results of imaging studies and treatment implications. Arch. Neurol..

[70] Harney-Delehanty B., Armeli S., Tennen H. (2026). Family history of alcohol use disorder and stress-reactivity. Anxiety Stress Coping.

[71] Yang S., Tong L., Zhang L., Cui R., Lyu X., He L., Liu H. (2026). Naltrexone treatment improves anxiety-and depression-like behavior in alcohol-exposed mice. Behav. Pharmacol..

[72] Zech J.M., Patel T.A., Cougle J.R. (2025). Prevalence and correlates of alcohol, cannabis, and tobacco use disorder in DSM-5 generalized anxiety disorder: Findings from the National Epidemiologic Survey on Alcohol and Related Conditions-III. J. Anxiety Disord..

[73] Meredith L.R., Baskerville W.A., Lee C., Grodin E.N., Wassum K.M., Ray L.A. (2025). Influence of real-world cue exposure and mood states on drinking: Testing neurobiological models of alcohol use disorder. Psychopharmacology.

[74] Yoon G., Pittman B., Ralevski E., Petrakis I.L., Krystal J.H. (2025). Antidepressant efficacy of ketamine plus naltrexone for major depression comorbid with alcohol use disorder: A randomized controlled trial. Int. J. Neuropsychopharmacol..

[75] Henricks A.M., Berger A.L., Lugo J.M., Baxter-Potter L.N., Bieniasz K.V., Petrie G., Sticht M.A., Hill M.N., McLaughlin R.J. (2017). Sex-and hormone-dependent alterations in alcohol withdrawal-induced anxiety and corticolimbic endocannabinoid signaling. Neuropharmacology.

[76] Laniepce A., Cabé N., André C., Bertran F., Boudehent C., Lahbairi N., Maillard A., Mary A., Segobin S., Vabret F. (2020). The effect of alcohol withdrawal syndrome severity on sleep, brain and cognition. Brain Commun..

[77] Shen G., Chen Y.-H., Wu Y., Jiahui H., Fang J., Jiayi T., Yimin K., Wang W., Liu Y., Wang F. (2024). Exploring core symptoms of alcohol withdrawal syndrome in alcohol use disorder patients: A network analysis approach. Front. Psychiatry.

[78] Hill K., Piercey C., Karoly H.C., Smith K.E. (2026). Kava (*Piper methysticum*) consumption patterns and conceptualizations: Results from an online survey. Subst. Abus. Treat. Prev. Policy.

[79] Pont-Fernandez S., Kheyfets M., Rogers J.M., Smith K.E., Epstein D.H. (2023). Kava (*Piper methysticum*) in the United States: The quiet rise of a substance with often subtle effects. Am. J. Drug Alcohol Abus..

[80] Lum A., Skelton E., McCarter K.L., Handley T., Judd L., Bonevski B. (2020). Smoking cessation interventions for people living in rural and remote areas: A systematic review protocol. BMJ Open.

[81] Buettner-Schmidt K., Miller D.R., Maack B. (2019). Disparities in rural tobacco use, smoke-free policies, and tobacco taxes. West J. Nurs. Res..

[82] Bittencourt L., Rubenstein D., Noonan D., McClernon F.J., Carroll D.M. (2024). Smoking quit attempts and associated factors among rural adults who smoke daily in the United States. Nicotine Tob. Res..

[83] Cornelius M.E. (2023). Tobacco product use among adults–United States, 2021. MMWR Morb. Mortal. Wkly. Rep..

[84] Wang T.W. (2018). Tobacco product use among adults—United States, 2017. MMWR Morb. Mortal. Wkly. Rep..

[85] Birdsey J. (2023). Tobacco product use among US middle and high school students—National Youth Tobacco Survey, 2023. MMWR Morb. Mortal. Wkly. Rep..

[86] Xu X., Bishop E.E., Kennedy S.M., Simpson S.A., Pechacek T.F. (2015). Annual healthcare spending attributable to cigarette smoking: An update. Am. J. Prev. Med..

[87] Nargis N., Hussain A.K.M.G., Asare S., Xue Z., Majmundar A., Bandi P., Islami F., Yabroff K.R., Jemal A. (2022). Economic loss attributable to cigarette smoking in the USA: An economic modelling study. Lancet Public Health.

[88] Mamallapalli J., Henderson A., Berthold E., Yoon S.L., Ray D., Lowe M., Grundmann O. (2025). Prevalence and use patterns of kava (*Piper methysticum*) in a US nationally representative sample. J. Ethnopharmacol..

[89] Bian T., Lynch A., Ballas K., Mamallapalli J., Freeman B., Scala A., Wang Y., Traboulsi H., Chellian R.K., Fagan A. (2024). Flavokavains A-and B-Free Kava Enhances Resilience against the Adverse Health Effects of Tobacco Smoke in Mice. ACS Pharmacol. Transl. Sci..

[90] Wang Y., Hashemian T., Bian T., Reznikov L.R., Bruijnzeel A.W., Lin T., Xing C. (2025). AB-Free Kava Suppresses Tobacco Smoke-Induced CREB Phosphorylation in the Mouse Cerebellum. Phytomed. Plus.

[91] Xing C., Malaty J., Malham M.B., Orlando F.A., Lynch A., Huo Z., François M., Firpi-Morell R., Fisher C.L., Christou D.D. (2024). The potential of AB-free kava in enabling tobacco cessation via management of abstinence-related stress and insomnia: Study protocol for a randomized clinical trial. BMC Complement Med. Ther..

[92] Imtiaz S., Shield K.D., Fischer B., Elton-Marshall T., Sornpaisarn B., Probst C., Rehm J. (2020). Recent changes in trends of opioid overdose deaths in North America. Subst. Abus. Treat. Prev. Policy.

[93] Florence C., Luo F., Rice K. (2021). The economic burden of opioid use disorder and fatal opioid overdose in the United States, 2017. Drug Alcohol Depend..

[94] Stevinson C., Huntley A., Ernst E. (2002). A systematic review of the safety of kava extract in the treatment of anxiety. Drug Saf..

[95] Anke J., Ramzan I. (2004). Pharmacokinetic and pharmacodynamic drug interactions with Kava (*Piper methysticum* Forst. f.). J. Ethnopharmacol..

[96] Gurley B.J., Gardner S.F., Hubbard M.A., Williams D.K., Gentry W.B., Khan I.A., Shah A. (2005). In vivo effects of goldenseal, kava kava, black cohosh, and valerian on human cytochrome P450 1A2, 2D6, 2E1, and 3A4/5 phenotypes. Clin. Pharmacol. Ther..

[97] Teschke R., Lebot V. (2011). Proposal for a kava quality standardization code. Food Chem. Toxicol..

